# Decreased atrioventricular plane displacement after acute myocardial infarction yields a concomitant decrease in stroke volume

**DOI:** 10.1152/japplphysiol.00480.2019

**Published:** 2019-12-19

**Authors:** J. Berg, R. Jablonowski, D. Nordlund, S. Kopic, S. Bidhult, C. G. Xanthis, M. Saeed, K. Solem, H. Arheden, M. Carlsson

**Affiliations:** ^1^Lund University, Faculty of Medicine, Department of Clinical Sciences Lund, Clinical Physiology, Lund, Sweden; ^2^Department of Radiology and Biomedical Imaging, University of California, San Francisco, California; ^3^Syntach AB, Lund, Sweden

**Keywords:** AMI, animal model, AVPD, cardiac failure, ischemia-reperfusion

## Abstract

Acute myocardial infarction (AMI) can progress to heart failure, which has a poor prognosis. Normally, 60% of stroke volume (SV) is attributed to the longitudinal ventricular shortening and lengthening evident in the atrioventricular plane displacement (AVPD) during the cardiac cycle, but there is no information on how the relationship changes between SV and AVPD before and after AMI. Therefore, the aim of this study was to determine how SV depends on AVPD before and after AMI in two swine models. Serial cardiac magnetic resonance imaging was carried out before and 1–2 h after AMI in a microembolization model (*n* = 12) and an ischemia-reperfusion model (*n* = 14). A subset of pigs (*n* = 7) were additionally imaged at 24 h and at 7 days. Cine and late gadolinium enhancement images were analyzed for cardiac function, AVPD measurements and infarct size estimation, respectively. AVPD decreased (*P* < 0.05) in all myocardial regions after AMI, with a concomitant SV decrease (*P* < 0.001). The ischemia-reperfusion model affected SV to a higher degree and had a larger AVPD decrease than the microembolization model (−29 ± 14% vs. −15 ± 18%; *P* < 0.05). Wall thickening decreased in infarcted areas (*P* < 0.001), and A-wave AVPD remained unchanged (*P* = 0.93) whereas E-wave AVPD decreased (*P* < 0.001) after AMI. We conclude that AVPD is coupled to SV independent of infarct type but likely to a greater degree in ischemia-reperfusion infarcts compared with microembolization infarcts. AMI reduces diastolic early filling AVPD but not AVPD from atrial contraction. These findings shed light on the physiological significance of atrioventricular plane motion when assessing acute and subacute myocardial infarction.

**NEW & NOTEWORTHY** The link between cardiac longitudinal motion, measured as atrioventricular plane displacement (AVPD), and stroke volume (SV) is investigated in swine after acute myocardial infarction (AMI). This cardiac magnetic resonance study demonstrates a close coupling between AVPD and SV before and after AMI in an experimental setting and demonstrates that this connection is present in ischemia-reperfusion and microembolization infarcts, acutely and during the first week. Furthermore, AVPD is equally and persistently depressed in infarcted and remote myocardium after AMI.

## INTRODUCTION

Heart failure (HF) is a major global health burden, and an estimated 6.5 million adults are suffering from this condition in the United States alone (3). One of the most common causes of HF is acute myocardial infarction (AMI), and patients with HF due to AMI have decreased longitudinal ventricular function (2, 37). The most common type of AMI is an infarction from an acute or rapidly aggravated obstruction of a coronary artery (41, 49). Microembolization (ME) infarction due to disseminated microemboli from a thrombus partly disintegrated during catheter intervention differs in category and results in another form of infarct pattern. A total obstruction of a coronary artery yields an infarct that is in most cases homogeneous, whereas the ME injury results in small and patchy islands of infarction in the downstream myocardium (11).

Longitudinal shortening of the ventricles, also called mitral annular plane systolic excursion, is essential for normal pumping and filling (20). It can be measured as the atrioventricular (AV) plane displacement (AVPD) with ultrasound (24) or cardiac magnetic resonance imaging (CMR) (13). In humans, ~60% of the left ventricular (LV) stroke volume (SV) is attributed to the longitudinal ventricular shortening in systole (13). Conversely, the longitudinal lengthening in diastole results in a scooping of the AV plane over atrial blood, resulting in diastolic filling (12). To this end, it is important to know *1*) the extent to which different myocardial ischemic injuries alter AVPD and SV and *2*) how the relationship between AVPD and SV changes with different types of myocardial injury.

We hypothesized that AMIs after ischemia-reperfusion (I/R) and ME affect the AVPD differently. To the best of our knowledge, the relationship between SV and AVPD has not previously been serially investigated. Therefore, the aims of this CMR study were to determine *1*) the relationship between AVPD and SV before and after AMI, *2*) whether the relationship between AVPD and SV differs between I/R and ME swine AMI models, and *3*) how diastolic longitudinal function changes after AMI.

## MATERIALS AND METHODS

### Animal Models

**Fig. 1.**
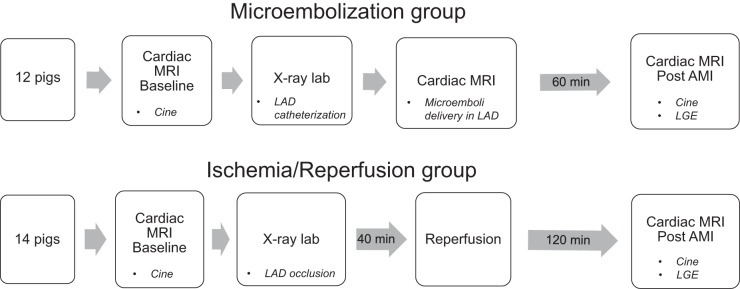
Study protocol showing the workflow for the Ischemia-Reperfusion and Microembolization groups. AMI, acute myocardial infarction; LAD, left anterior descending artery; LGE, late gadolinium enhancement; MRI, magnetic resonance imaging.

This study was performed in agreement with the *Guide for the Care and Use of Laboratory Animals* (18) and with approval from the Swedish Agricultural Board and the Institutional Animal Care and Use Committee at the University of California, San Francisco. Two infarct models were used in this swine study: *1*) the ischemia-reperfusion model: pigs with a balloon occlusion-reperfusion injury with 40 min of ischemia before reperfusion (27) and *2*) the microembolization model: pigs with intracoronary injection of microemboli (10, 11). An overview of the study protocol is shown in Fig. 1, and the appendix provides anesthetic details.

#### Ischemia-reperfusion.

Fourteen healthy farm pigs (~40 kg) were subjected to left anterior descending artery (LAD) occlusion for 40 min, permitting an infarct evolution of ~50% of the myocardium at risk (22). Occlusion was placed distal to the first diagonal (D1) and proximal to the second diagonal (D2). Short-axis and long-axis cine CMR images were acquired at baseline and 2 h after AMI for assessment of LV function and volumetric analysis. At the same image planes, late gadolinium enhancement (LGE) was performed for infarct size estimation. Additionally, a subset of animals (*n* = 7) were imaged at 24 h and 7 days to monitor the changes in function during the first week after AMI.

#### Microembolization.

This model has previously been described in detail (10, 11). Briefly, 12 healthy farm pigs (~33 kg) underwent an intracoronary injection of one of two sizes of embolic material (40–120 µm, 250,000 microsphere count or 100–300 µm, 7,200 microsphere count; 6 pigs with each microembolic size) distal to the first diagonal branch and were imaged 1 h after injection. Coronary catheterization, intervention, and imaging were performed in a hybrid X-ray/MR suite that included an X-ray system (Integris V5000; Philips Medical Systems, Best, The Netherlands) and an MR imager (Achieva I/T; Philips Medical Systems).

### CMR Image Parameters

#### Ischemia-reperfusion.

Short- and long-axis cine images were acquired at baseline and 2 h after reperfusion. Two different scanners were used for this group. A Philips Achieva 1.5T was used for 7 of 14 animals with the following image parameters: steady-state free precession (SSFP) sequence: repetition time (TR) 3 ms, echo time (TE) 1.5 ms, flip angle 60°, and slice thickness 8 mm with no slice gap. A Siemens Aera 1.5T was used for the other seven animals with the following image parameters; SSFP sequence: TR 2.7 ms, TE 1.2 ms, flip angle 60°, and slice thickness 8 mm with no slice gap. LGEs at the same image planes were acquired for estimation of infarct size. The inversion time was chosen to null remote myocardium. LGE parameters for the Philips Achieva 1.5T scanner were TR 4.1 ms, TE 1.3 ms, flip angle 15°, field of view 122 × 122 mm, pixel size 1.52 × 1.52 mm, no slice gap. LGE parameters for the Siemens Aera 1.5T scanner were TR 2.8 ms, TE 1.2 ms, flip angle 50°, field of view 159 × 154 mm, pixel size 1.41 × 1.41 mm, no slice gap.

#### Microembolization.

Cine MRI (Philips Achieva 1.5T) was performed at baseline and 1 h after AMI with a SSFP sequence: TR 3.5 ms, TE 1.8 ms, flip angle 70°, slice thickness 10 mm with no slice gap; field of view = 25 × 25 cm, matrix size = 160 × 152, heart phases = 16. Short-axis and long-axis cine images were acquired at baseline and 1–2 h after ME. LGE images were acquired in short- and long-axis views through the LV with an inversion-recovery gradient-echo sequence: TR 5.2 ms, TE 1.5 ms, flip angle 15°, pixel size 1.02 × 1.60 mm, no slice gap. The inversion time was chosen to null remote myocardium.

#### CMR parameters: summary of differences.

The main differences in the protocols were that *1*) the Microembolization group were imaged with two perpendicular horizontal and vertical long-axis views for both cine and LGE, whereas the Ischemia-Reperfusion group were imaged with 2-, 3-, and 4-chamber long-axis views. This difference reduces the number of annotation points for AVPD calculations from six in the Ischemia-Reperfusion group to four in the Microembolization group. *2*) An inversion-recovery gradient-recalled echo sequence for the LGE images was used for the Microembolization group and 7 of 14 animals in the Ischemia-Reperfusion group (28). In the other seven animals of the Ischemia-Reperfusion group, LGE was acquired with a phase-sensitive inversion-recovery single-shot SSFP sequence in free breathing with motion correction (30). *3*) On the same cine SSFP sequence, the slice thickness was 10 mm and 8 mm in the Microembolization and Ischemia-Reperfusion groups, respectively.

### Image Postprocessing and Analysis

#### Cardiac parameters.

The images were analyzed with the postprocessing software Segment v2.2 (Medviso, Lund, Sweden) (23). The endocardium and epicardium were manually delineated in the short-axis end-systolic and end-diastolic images to determine LV mass (LVM) and volumes, including SV. Delineations from midventricular slices were also used for determining regional wall thickening (14). The delineations were done in consensus between two investigators. Cardiac output (CO) was determined as SV × heart rate. LVM was calculated automatically from short-axis slices with a myocardial specific density of 1.05 g/cm^3^.

#### AVPD.

**Fig. 2.**
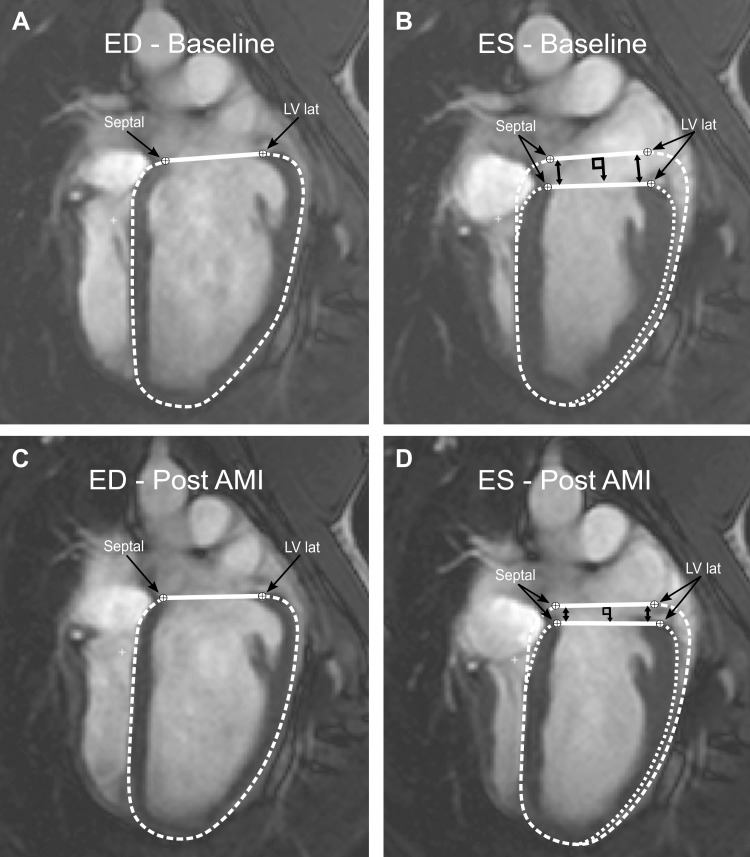
Illustration of how the atrioventricular (AV) plane displacement (AVPD) was measured in a 4-chamber long-axis view of the heart. The difference in longitudinal shortening between baseline and after acute myocardial infarction (AMI) in the same animal is shown. *A*: end diastole (ED) at baseline, where the left ventricular (LV) contours are shown. The single arrows show the septal and lateral annotation points in ED, which defines the AV plane, shown as a solid line. *B*: end systole (ES) at baseline. The LV contours from ED are superimposed over the LV contours in ES. The black double arrows show the distance the AV plane has moved. The distance is measured perpendicular from the AV plane defined in ED. *C*: ED after AMI. *D*: ES after AMI. The LV contours from ED are superimposed over the ES image, and the AVPD is markedly decreased from baseline.

AVPD annotation points were placed in each time frame during the cardiac cycle in all different available long-axis slices with a semiautomatic AVPD tracking algorithm developed in-house, with manual corrections (44). The annotation points were selected to be at the highest point of the myocardium, at the level of the mitral valve plane, as previously described (13), and followed throughout the cardiac cycle. Figure 2 illustrates the method of AVPD measurement.

#### Anteroseptal and remote myocardium.

The individual AVPD points encircling the LV were divided into anteroseptal (AVPD_antsep_) and remote (AVPD_remote_) regions. AVPD_antsep_ included the two septal points from the 3-chamber and 4-chamber views and the anterior point from the 2-chamber view. AVPD_remote_ included the lateral points from the 3-chamber and 4-chamber views and the posterior point from the 2-chamber view. The mean longitudinal distances of the separate points were calculated for the AVPD_antsep_ and AVPD_remote_ zones. The wall thickening analyses were divided into anteroseptal (WT_antsep_) and remote (WT_remote_) halves to match the regional AVPD measurements.

#### Infarct size.

LGE images were used to estimate infarct size in the Ischemia-Reperfusion group with the “EWA” algorithm with manual corrections (17) at 2 h after reperfusion. LGE images in the Microembolization group were analyzed with a thresholding method of the signal intensity 3 standard deviations higher than remote myocardium (15). Both methods have previously been validated against ex vivo histochemical staining in ischemia-reperfusion (17) and microembolization infarct models (11, 15).

#### E-wave and A-wave AVPD.

The AVPD was divided into the early diastolic filling phase (E-wave AVPD) and the atrial contraction phase (A-wave AVPD) with the diastasis as the dividing point, since this is the equilibrium state of the cardiac cycle (50). Only animals with an identifiable diastasis in both baseline and AMI cine images were included for this analysis. E-wave AVPD was determined as the distance from the diastasis level to the most apical position the AV plane reached and A-wave AVPD as the distance from the diastasis level to the most basal position the AV plane reached.

### Statistics

Paired and unpaired two-tailed Student’s *t* tests were used to compare means between variables between time points and between groups, respectively. Within the same animal the same myocardial region (e.g., AVPD_antsep_) was compared by paired *t* test with the same region at 2 h, whereas comparisons between different regions (e.g., AVPD_antsep_ with AVPD_remote_) within the same animal were made by unpaired *t* test. Serial measurements were compared by repeated-measures ANOVA. All statistical analyses were performed with SPSS Statistics v.25 (IBM, Armonk, NY) and GraphPad Prism v.7.04 (GraphPad Software, La Jolla, CA), and differences with a *P* value < 0.05 were considered significant. The results for each group are presented as means ± SD and change in percentage units.

## RESULTS

### Cardiac Parameters

At baseline, SV, end-diastolic volume (EDV), and end-systolic volume (ESV) were larger in the Ischemia-Reperfusion group compared with the Microembolization group (*P* < 0.01 for all). AVPD measures, CO, heart rate, and ejection fraction (EF) did not differ between the groups at baseline (*P* = not significant). Cardiac parameters at baseline and after AMI are shown in Table 1.

**Table 1.** Baseline and post-AMI data in Ischemia-Reperfusion and Microembolization groups

	Ischemia-Reperfusion (*n* = 14)			Microembolization (*n* = 12)		
Parameters	Baseline	Post AMI	Δ % change	Baseline	Post AMI	Δ % change
LV AVPD, mm	10.0 ± 1.8	7.2 ± 2.1***	−29 ± 14	9.3 ± 1.7	7.7 ± 1.5*	−15 ± 18‡
LV AVPD anteroseptal, mm	9.8 ± 1.3	6.7 ± 2.0***	−32 ± 15	8.9 ± 1.7	7.2 ± 1.6**	−17 ± 19‡
LV AVPD remote, mm	11.3 ± 2.4	8.5 ± 2.1***	−25 ± 11	10.5 ± 1.9	8.7 ± 1.7**	−16 ± 17
LV stroke volume, mL	48 ± 11	33 ± 10***	−33 ± 13	37 ± 5‡‡	26 ± 4***	−31 ± 11
Basal epicardial area, cm^2^	27.9 ± 1.9	26.7 ± 1.9**	−4 ± 6	24.9 ± 1.2	25.7 ± 1.7*	3 ± 5‡‡‡
Long. contribution to SV, %	59 ± 13	59 ± 9	0 ± 10	62 ± 15	78 ± 14***	15 ± 11‡‡‡
LV ejection fraction, %	48 ± 6	36 ± 7***	−12 ± 6	49 ± 5	31 ± 5***	−18 ± 6‡
LV end-diastolic volume, mL	100 ± 14	91 ± 18***	−10 ± 9	76 ± 9‡‡‡	83 ± 10***	9 ± 6‡‡‡
LV end-systolic volume, mL	52 ± 7	58 ± 12*	12 ± 15	39 ± 6‡‡‡	58 ± 9***	49 ± 19‡‡‡
Heart rate, beats/min	80 ± 12	98 ± 34	23 ± 40	91 ± 16	80 ± 9*	−10 ± 17‡
Cardiac output, L/min	3.9 ± 1.0	3.0 ± 0.9*	−17 ± 30	3.4 ± 0.8	2.1 ± 0.4***	−37 ± 19
Wall thickening anteroseptal, %	48 ± 13	16 ± 12***	−32 ± 15	36 ± 9	23 ± 9***	−13 ± 7‡‡‡
Wall thickening remote, %	47 ± 15	52 ± 18	5 ± 16	38 ± 11	38 ± 13	1 ± 12
Infarct size, % LV		26 ± 11			8 ± 2‡‡‡	

Values are means ± SD for *n* animals. AMI, acute myocardial infarction; AVPD, atrioventricular plane displacement; LV, left ventricular; SV, stroke volume.

* *P* ≤ 0.05,

** *P* ≤ 0.01,

*** *P* ≤ 0.001 compared with baseline.

‡ *P* ≤ 0.05,

‡‡ *P* ≤ 0.01,

‡‡‡ *P* ≤ 0.001 compared with the Ischemia-Reperfusion group.

Acutely after AMI, EDV decreased (−10 ± 9%; *P* < 0.001) in the Ischemia-Reperfusion group and increased (9 ± 6%; *P* < 0.001) in the Microembolization group. ESV increased significantly in the Ischemia-Reperfusion group (12 ± 15%; *P* < 0.05) and even more in the Microembolization group (49 ± 19%; *P* < 0.001). Heart rate increased in the Ischemia-Reperfusion group (23 ± 40%) and decreased in the Microembolization group (−10 ± 17%) (*P* < 0.05 for both). Thus the decreased EDV in the Ischemia-Reperfusion group is most likely attributable to the substantial increase in heart rate. At the global level, CO decreased differently in Ischemia-Reperfusion and Microembolization groups compared with baseline [−17 ± 30% (*P* < 0.05) and −37 ± 19% (*P* < 0.001), respectively]. EF also decreased in both groups compared with baseline (*P* < 0.001 for both) but to a lesser extent in the Ischemia-Reperfusion group than the Microembolization group (−12 ± 6% vs. −18 ± 6%; *P* = 0.018).

### SV and AVPD

**Fig. 3.**
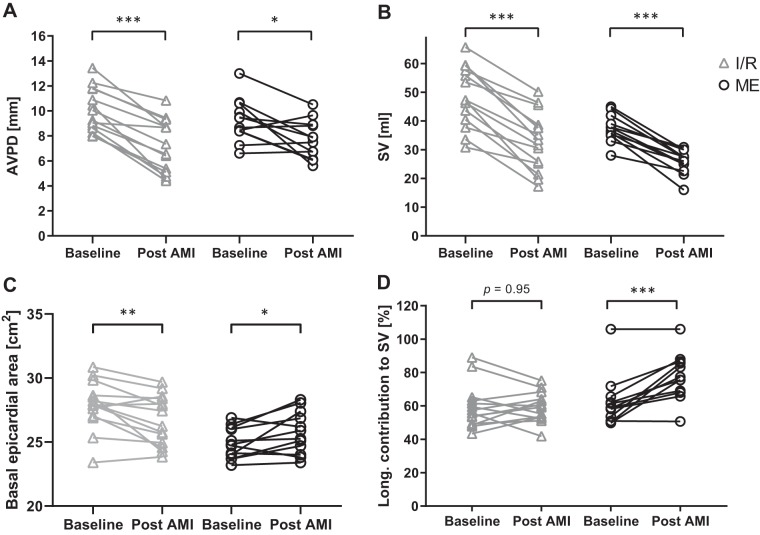
Atrioventricular plane displacement (AVPD), stroke volume (SV), basal epicardial area, and longitudinal contribution to SV before and 1–2 h after acute myocardial infarction (AMI). *A* and *B*: the Ischemia-Reperfusion (I/R) group displayed a larger decrease in AVPD after AMI (*P* < 0.001) than the Microembolization (ME) group (*P* < 0.05) (*A*), whereas SV decreased to a similar extent in both groups (*P* < 0.001 for both, *B*). *C*: the basal epicardial area decreased in the I/R group (*P* < 0.01) and increased in the ME group (*P* < 0.05). *D*: these variables result in that longitudinal contribution to SV was unchanged (*P* = 0.95) and increased (*P* < 0.001) in the I/R and ME groups, respectively. *P < 0.05, **P < 0.01, ***P < 0.001 compared with baseline.

The induced infarctions caused a larger decrease in AVPD in the Ischemia-Reperfusion group (−29 ± 14%) compared with the Microembolization group (−15 ± 18%; *P* < 0.05; Fig. 3*A*), where the magnitudes of SV decrease were similar (−33 ± 13% and −31 ± 11%, respectively; Fig. 3*B*). The basal epicardial slice area decreased in the Ischemia-Reperfusion group and, on the contrary, increased in the Microembolization group (−4 ± 6 cm^2^ vs. 3 ± 5 cm^2^; Fig. 3*C*), in parallel with EDV. This caused the longitudinal contribution to SV to remain unchanged in the Ischemia-Reperfusion group (0 ± 10%; *P* = 0.95) and increase in the Microembolization group (15 ± 11%; *P* < 0.001; Fig. 3*D*) after AMI.

**Fig. 4.**
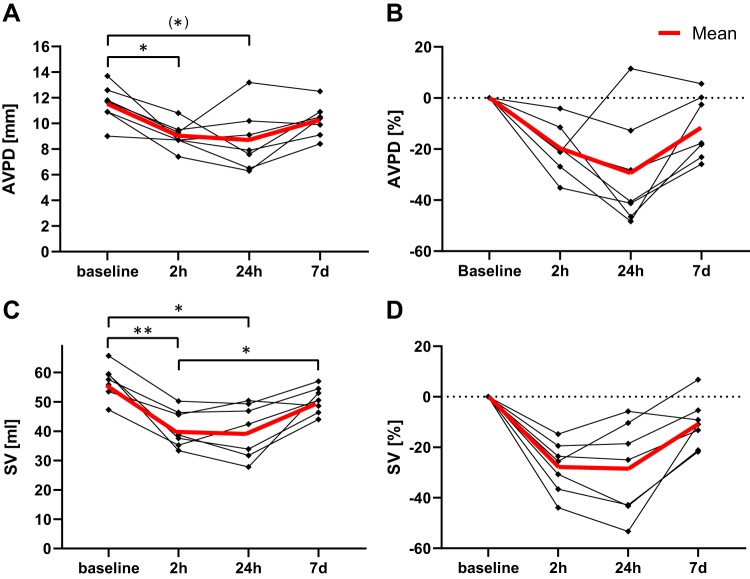
The chronological evolutions of atrioventricular plane displacement (AVPD) and stroke volume (SV) were investigated in a subset of animals in the Ischemia-Reperfusion group (*n* = 7) during the first week. AVPD (*A* and *B*) and SV (*C* and *D*) show a similar initial decrease during the first day after ischemia, which recuperates to near-baseline values at *day 7*. ^(^*^)^*P* = 0.084, *P < 0.05, **P < 0.01.

The chronological evolution during the first week after AMI was concordant between SV and AVPD, namely, a continued decrease at 24 h (*P* < 0.01 for SV and *P* < 0.05 for AVPD) and recovery to near-baseline values at *day 7* (Fig. 4).

**Fig. 5.**
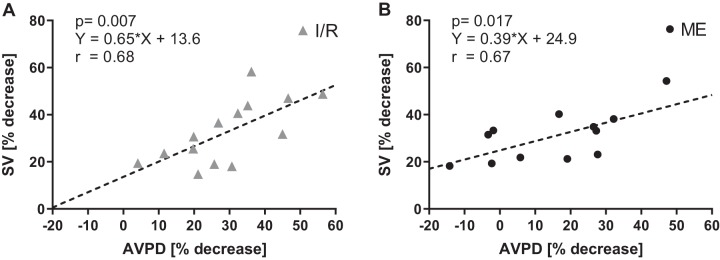
The relationship between the individual % change in both stroke volume (SV) and atrioventricular plane displacement (AVPD) before and after acute myocardial infarction (AMI) in the Ischemia-Reperfusion (I/R) group (*A*) and the Microembolization (ME) group (*B*). The higher slope of regression in the I/R group vs. the ME group (0.65 × *x* + 13.6 vs. 0.39 × *x* + 24.9) indicates that there is greater impact on longitudinal wall motion in I/R injury compared with ME injury.

Individual changes of both SV and AVPD reached a steeper slope of regression in the Ischemia-Reperfusion group compared with the Microembolization group (0.65 × *x* + 13.6 vs. 0.39 × *x* + 24.9; see Fig. 5), but the regression coefficients did not differ (*r* = 0.68 vs. *r* = 0.67). Both groups combined reached a correlation of *r* = 0.57.

### AVPD: Anteroseptal vs. Remote Myocardium

**Fig. 6.**
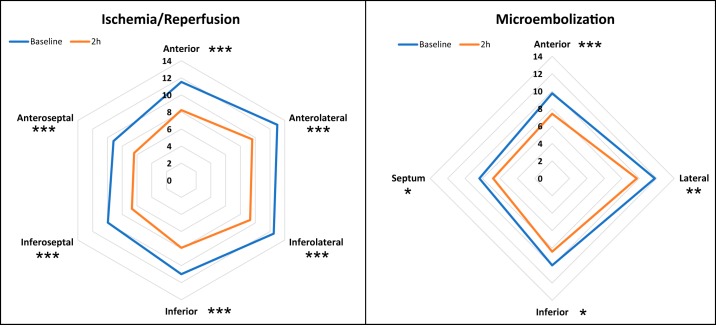
Radar charts displaying the average value for the individual atrioventricular plane displacement (AVPD) points before and after infarction for the Ischemia-Reperfusion (I/R) and Microembolization (ME) groups. The reduction in AVPD was more pronounced in the I/R group compared with the ME group but was homogeneous throughout the myocardium in both groups. The number of annotation points differs from 6 in the I/R group to 4 in the ME group because of different imaging protocols. *P < 0.05, **P < 0.01, ***P < 0.001 compared with baseline.

**Fig. 7.**
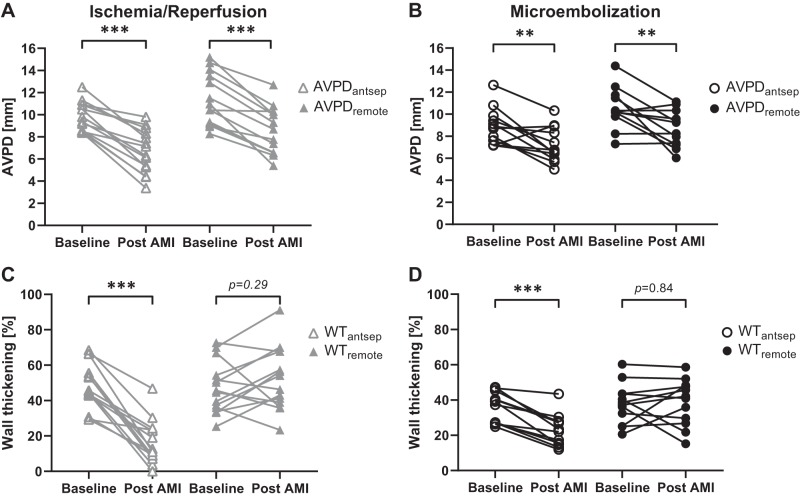
Atrioventricular plane displacement (AVPD, *A* and *B*) and wall thickening (WT, *C* and *D***)** analyses for anteroseptal (antsep) and remote myocardium in the Ischemia-Reperfusion (I/R, triangles) and Microembolization (ME, circles) groups. There was a homogeneous decrease in AVPD in both anteroseptal and remote regions (*P* < 0.01 for all). The infarct caused depressed wall thickening in the anteroseptal region in both groups (*P* < 0.001). Although hypercontractile myocardium was seen in some animals in the remote regions in the I/R group, neither of the groups reached significance. **P < 0.01, ***P < 0.001 compared with baseline.

Reductions in individual AVPD points were more prominent in the Ischemia-Reperfusion group compared with the Microembolization group, but no single point showed a considerable deviation from the mean change in either group (Fig. 6). The AVPD reduction from baseline to after AMI was not different in the anteroseptal (AVPD_antsep_) compared with remote (AVPD_remote_) myocardium in the Ischemia-Reperfusion group (−32 ± 15% vs. −25 ± 11%; *P* = 0.08) or in the Microembolization group (−17 ± 19% vs. −16 ± 17%; *P* = 0.71; Fig. 7, *A* and *B*). The infarcts caused depressed wall thickening in the anteroseptal region to a greater degree in the Ischemia-Reperfusion group (−32 ± 15%) compared with the Microembolization group (−13 ± 7%; *P* < 0.001; Fig. 7, *C* and *D*). Unlike the Microembolization group, hypercontractile myocardium in remote regions was seen in some animals in the Ischemia-Reperfusion group but did not reach statistical significance (*P* = 0.29).

### Infarct Size and AVPD

**Fig. 8.**
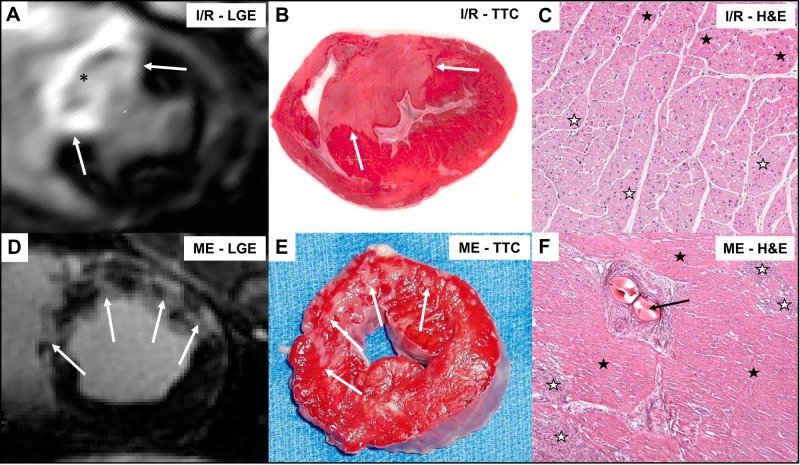
Example of infarct extent (white arrows) with in vivo late gadolinium enhancement (LGE) images (*A* and *D*) and corresponding ex vivo triphenyltetrazolium chloride (TTC) staining (*B* and *E*) for the Ischemia-Reperfusion (I/R) group (*top*) and the Microembolization (ME) group (*bottom*). LGE with early hypoenhanced core (black asterisk) representing a homogeneous infarct with microvascular obstruction core (*A*) and a patchy enhancement pattern (*D*) displays differences in infarct distribution and severity between the 2 groups. *C* and *F*: 2 illustrative examples of histological staining with hematoxylin and eosin (H&E) showing infarcted tissue (white stars) and intact myocytes (black stars) for an ischemia-reperfusion infarct (*C*) and a microembolization infarct (*F*). *F* (ME group) shows an arteriole with trapped microemboli (black arrow) and infarct islets (white stars) and was taken 1 wk after infarction. The illustrative biopsy in *C* shows a continuous shift from intact myocytes (black stars) toward stainless, damaged myocytes (white stars). The biopsy is from another set of animals from our group, performed with the same infarct protocol and acquired 4 h after reperfusion.

**Fig. 9.**
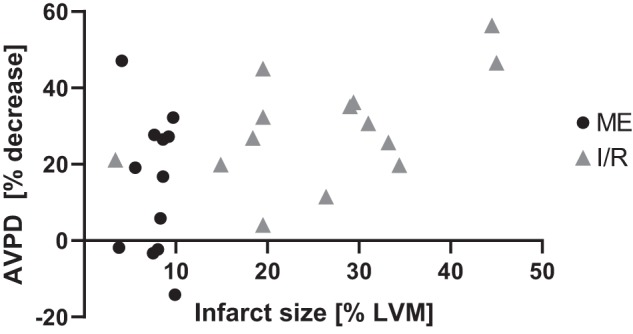
Percent decrease in atrioventricular plane displacement (AVPD) before and 2 h after acute myocardial infarction plotted against infarct size in % of left ventricular mass (LVM) for the Ischemia-Reperfusion (I/R) and Microembolization (ME) groups. There is moderate evidence for the I/R group toward larger infarcts yielding larger decreases in AVPD (*r* = 0.53, *P* *=* 0.05). How microembolization infarcts affected AVPD varied greatly, as shown by the large vertical spread with simultaneous small variance in infarct sizes.

Illustrative examples of infarcts from the two groups are shown in Fig. 8. The Ischemia-Reperfusion group had larger infarcts in total (26 ± 11% LVM) and a larger variance within the group compared with the infarcts in the Microembolization group (8 ± 2% LVM; Fig. 9). The correlation between infarct size and the percent decrease in AVPD was not significant in the Microembolization group (*r* = 0.16, *P* = 0.61). However, larger infarcts tended to yield larger AVPD decreases in the Ischemia-Reperfusion group (*r* = 0.53, *P* *=* 0.05; Fig. 9).

### E-Wave and A-Wave AVPD

**Fig. 10.**
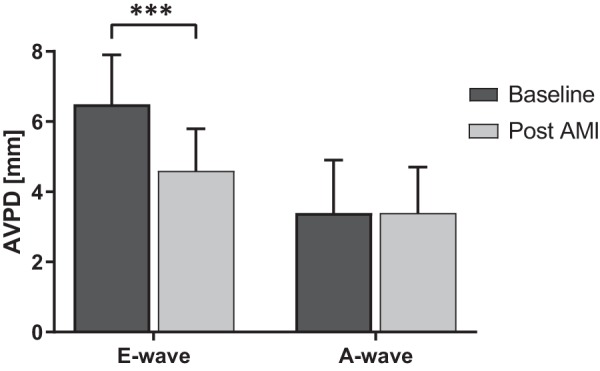
The atrioventricular plane displacement (AVPD) values from 14 of 26 animals (*n* = 7 Microembolization group, *n* = 7 Ischemia-Reperfusion group) in which the diastasis was clearly identified were pooled and examined regarding diastolic filling phase (E-wave AVPD) and late atrial contraction phase (A-wave AVPD). Longitudinal movement during E wave, but not A wave, decreased after acute myocardial infarction (AMI). ***P < 0.001 compared with baseline.

The diastasis in the cardiac cycle was successfully identified both at baseline and after AMI in 14 of 26 animals (7 from Microembolization group, 7 from Ischemia-Reperfusion group), and these animals were pooled to reach a larger sample size. Identification of diastasis was not performed in animals with heart rates > 90 beats/min because of merging of the early diastolic movement and atrial contraction. The mean longitudinal shortening for E-wave AVPD decreased significantly (from 6.5 ± 1.4 mm to 4.6 ± 1.2 mm; *P* < 0.001), whereas the longitudinal shortening for A-wave AVPD remained unchanged after AMI (from 3.4 ± 1.5 mm to 3.4 ± 1.3 mm; *P* = 0.93; Fig. 10).

## DISCUSSION

We demonstrated how LV SV is concomitantly decreased with LV AVPD after AMI in two different myocardial infarct models and monitored the pathophysiological responses during the first week. Importantly, this experimental setting with serial CMR imaging before and after the ischemic injuries enabled us to link the change in SV to the change in AVPD, and we found that the relationship between SV and AVPD was unchanged by ischemia-reperfusion injury both acutely and over the first week after injury. The reduction in AVPD was larger with ischemia-reperfusion infarcts compared with microembolization infarcts, while SV was similarly decreased. Furthermore, the ischemia-reperfusion infarcts had greater impact on longitudinal wall motion than the patchy microembolization infarcts. These results show differences in the pathophysiological response of ventricular pumping between the two models of AMI. Of note, a similar decrease of AVPD was found around the whole LV in both groups, indicating that longitudinal motion is globally affected by a regional ischemic injury. AVPD during early diastolic filling (E wave) decreased after AMI compared with baseline, whereas AVPD during atrial contraction (A wave) was unchanged. Thus the relative involvement of atrial contraction to total AVPD increased after AMI.

### SV vs. AVPD

The results in our study are in line with previous clinical studies reporting that AVPD decreases after myocardial infarction (1, 6, 25, 37). This experimental study shows how the SV and related cardiac parameters change as a result of decreased AVPD after AMI, which is not possible to investigate in clinical studies, where a baseline examination is practically difficult. It also highlights that the repeated measures before and after infarction suggest a direct link between SV and AVPD on the individual level. Our SV and AVPD correlations are similar to the results of Carlhäll et al. with echocardiography in patients (*r* = 0.80) (8). SV and AVPD decreased acutely after AMI in a parallel fashion, especially in the infarcts of the Ischemia-Reperfusion group. The recovery of these parameters continued during the first week. The peak deterioration for both SV and AVPD occurred at 24 h after infarction and later recovered to a degree in which no significant differences were found compared with baseline values. This is in line with other studies that have shown a slow but significant recovery of cardiac function after AMI over days to weeks (9, 16, 29). The outlier in Fig. 4, which experienced increased longitudinal motion at 24 h, had a minor infarct size (<4% LVM).

The slope of regression was higher in the Ischemia-Reperfusion group compared with the Microembolization group, whereas the regression coefficients were similar. The higher the regression slopes, the greater the influence of the type of ischemia on wall motion and SV. This means that the ischemia-reperfusion infarcts have a greater impact on longitudinal motion than the patchy microembolization infarcts. The link between SV and AVPD is present in both groups, although the steeper slope of regression suggests slightly different pathophysiological mechanisms of the AMI models.

Earlier echocardiographic and ventriculographic studies comparing AVPD with EF showed strong correlation coefficients of 0.78–0.84 (45), 0.87 (1), and 0.95 (38). However, later studies have shown that these parameters are not interchangeable and seem to infer different prognostic information. Patients undergoing CMR who had a higher EF along with decreased AVPD had a decisively worse prognosis than those with low EF and normal AVPD. Investigators concluded that AVPD is the best prognostic marker for major adverse cardiac events (39) and mortality (40). This proposition is sound when considering that EF is normal in many forms of disease, such as with LV hypertrophy and HF with diastolic dysfunction and preserved EF. The AVPD, however, is dependent on both systolic and diastolic ventricular properties as well as the coupling with atrial function, which may explain why decreased longitudinal shortening has a strong prognostic value in patients.

### AVPD: Anteroseptal vs. Remote Myocardium

To investigate whether a regional AMI reduces the AVPD globally or only regionally, the AVPD points were first individually measured and then compared in the anteroseptal myocardium (AVPD_antsep_) and remote myocardium (AVPD_remote_). The individual AVPD points show a similar decrease encircling the LV after infarction in both groups and also show inherently greater lateral than septal AV plane movement. In anteroseptal and remote regions, the decrease in AVPD was significantly larger in the Ischemia-Reperfusion group than in the Microembolization group. All results combined suggest that AVPD is likely to be globally reduced after AMI. This has also recently been reported by Pahlm et al. in patients (*n* = 177) within a week after ST-elevation myocardial infarction (37).

Two patterns of wall thickening could be observed in remote myocardium of the Ischemia-Reperfusion group after AMI, namely, thickening and thinning (Fig. 7*C*; *P* = 0.29). However, no increased wall thickening was seen in the Microembolization group. The most likely reason for the different cardiac responses is the greater stress imposed on the heart in an occlusion-reperfusion event compared with microembolization. This would explain the slight (but not statistically significant) increase in percent wall thickening in remote regions in the Ischemia-Reperfusion group and also the opposite responses in heart rate.

There are many factors that could influence wall motion in the early phase (hours) after AMI, such as myocyte necrosis, cytokine-mediated inflammation, edema, and microvascular dysfunction (26). Furthermore, Stoylen attributed the globally decreased AV plane movement after an ischemic injury to anatomical factors, mainly because of the rigid annulus fibrosus, which connects all four cardiac valves and the myocardium. This prohibits independent regional shortening and reduces strain on surrounding myocytes, yielding compensatory contraction (47). Speculatively, the vast interconnection between the contracting myocardial meshwork in which the myocyte helical angle gradually changes from epi- to endocardium (33, 34, 36) is another likely contributor responsible for dysfunction of remote myocardium.

### Infarct Size and AVPD

Similarly to previous CMR studies (49a, 42), the late gadolinium-enhanced CMR images showed that the Ischemia-Reperfusion group had substantially larger infarct sizes, often with core microvascular obstruction zones, than the patchy microembolization infarcts, which might cause the larger reductions in AVPD in the Ischemia-Reperfusion group. Global LV impairment as reflected by decreased SV and CO was comparable despite the large difference in infarct sizes between the two groups.

Despite a small interval of infarct sizes in the Microembolization group, the AVPD reductions displayed large variations. The most likely mechanism behind such variations is the patchy nature and random distribution of microembolus infarcts (11, 35, 43), resulting in variable injuries on subendocardial longitudinal fibers. The heart rate response after MI was different between the groups. The decrease in heart rate in the Microembolization group and the increase in Ischemia-Reperfusion group may explain part of the difference in AVPD responses.

### E-Wave and A-Wave AVPD

The E-wave AVPD significantly decreased whereas the A-wave AVPD remained constant after AMI. The diastasis can be used as the reference point for the cardiac cycle as it represents the equilibrium volume (50). At this point, the AV plane motion dependent on diastolic ventricular myocardial suction of blood into the ventricle due to recoil has finished (E-wave AVPD). The A-wave AVPD depends on atrial myocardium contraction. Following this reasoning, the infarcts would only affect the E-wave AVPD since there is damage to the ventricular myocardium while the atrial myocardium is uninjured. This indicates that the atrial “kick” is maintained after infarction. These findings also confirm the clinical observations by Kranidis et al. (31).

The maximum distance that the AV plane travels during the cardiac cycle depends on ventricular contraction, relaxation, and coupling with atrial contraction. A decreased ventricular contraction after myocardial damage leads to reduced compression of myocardial titin and hence a reduced amount of potential energy stored in the “cardiac spring” as well as reduced stretch of the left atrium (19, 21). Consequently, the AV plane has traveled a lesser distance from equilibrium position at diastasis.

The SV entering the aorta generated by the LV is constantly greater than blood simultaneously entering the left atrium swept by systolic AVPD (Doppler S wave). This volume debt is accounted for by the epicardial crescent effect (48), which generates an additional inflow of blood through the atrium during diastolic filling (Doppler D wave) (5, 46). A maintained atrial contraction after infarction will aid the ventricle in late diastolic filling caused by additional ejection of atrial blood.

### Future Aspects

The significance of the AVPD in cardiac physiology and prognosis has captured the interest of many researchers in the last decades. With the advent of noninvasive CMR, the contribution of AV plane motion could be accurately and reputedly measured (12). The prognostic impact of long-axis LV measurements in larger patient cohort studies (20a, 39, 40) further adds to its possible usefulness as a research endpoint. The results of this study along with findings from the abovementioned studies combine to the hypothesis that longitudinal motion potentially could be a novel and innovative therapeutic target for future cardiac assist devices. A globally reduced longitudinal function after a regional infarct indicates that patients regardless of infarct type could benefit from an augmented long-axis function. Studies investigating the causality regarding whether an increased longitudinal motion increases cardiac performance are lacking and would therefore be highly interesting.

### Limitations

The results of the study should be interpreted in light of some limitations.

#### CMR protocol.

Two different MRI scanners were used. However, the scanners and sequences in this study are widely and clinically used. The anesthetic protocols slightly differed between the AMI models, which could alter the hemodynamic response. The reference in both models, however, was a baseline scan in relation to which the anesthesia protocol was unchanged. Different times to imaging after AMI (1–2 and 2 h) were utilized in the AMI groups. The differences in CMR protocols prevented blinding between the two groups during the analyses, which is a limitation of the study.

#### Infarct protocol.

The embolization particles may not exert or mimic the same pathological response in the myocardium as disseminated plaques and thrombus do. Infarctions were induced in the LAD territory for both ischemia-reperfusion and microembolization models. It is not known if these results can be transferred to ischemic injuries in other coronary territories.

#### Anesthesia.

The preconditioning effects of isoflurane could be different for the ischemia-reperfusion and microembolization models, and this would impact the results.

#### Load dependence.

Loading conditions could affect AVPD in parallel with ventricular contraction. Invasive blood pressure data were not systematically recorded. The one-third of animals with recorded blood pressure measurements did not deviate more than 10 mmHg from baseline to post-AMI imaging. However, carefully measuring longitudinal function in isolated and variable pre- and postloading conditions would be highly interesting and a quest for future studies.

#### E-wave and A-wave AVPD.

The number of animals for this analysis was small (14 out of 26), and the results should therefore be interpreted with slight reservation.

### Conclusions

AVPD is depressed after AMI and is linked to a decreased SV. The coupling between decreased AVPD and SV is present in microembolization infarcts and likely to a greater degree in ischemia-reperfusion infarcts and evolves in concordance during the first week after AMI. These findings shed light on the physiological significance of AV plane motion when assessing acute and subacute myocardial infarction.

## GRANTS

This work was supported by Hjärt-Lungfonden (Swedish Heart-Lung Foundation), Vetenskapsrådet (Swedish Research Council), and the Swedish Foundation for Strategic Research (SSF).

## DISCLOSURES

J. Berg and K. Solem are employed by Syntach AB, a med-tech company in Lund, Sweden. None of the other authors has any conflicts of interest, financial or otherwise, to disclose.

## AUTHOR CONTRIBUTIONS

J.B., M.S., K.S., H.A., and M.C. conceived and designed research; J.B., R.J., D.N., S.K., S.B., C.G.X., and M.S. performed experiments; J.B., M.S., and M.C. analyzed data; J.B., R.J., H.A., and M.C. interpreted results of experiments; J.B. prepared figures; J.B., R.J., and M.C. drafted manuscript; J.B., R.J., D.N., S.K., S.B., C.G.X., M.S., K.S., H.A., and M.C. edited and revised manuscript; J.B., R.J., D.N., S.K., S.B., C.G.X., M.S., K.S., H.A., and M.C. approved final version of manuscript.

## APPENDIX

### Ischemia-Reperfusion

Fourteen pigs (Landrace and Yorkshire, ∼40 kg) were premedicated with 0.5 mg/kg midazolam (Dormicum; Roche, Stockholm, Sweden) and 15 mL/kg ketamine (Ketaminol; Intervet, Dandery, Sweden), both intramuscular. The pigs were intubated with additional intravenous 2- to 3-mg propofol boluses if required for adequate relaxation. The anesthesia was maintained with isoflurane (Baxter Medical, Kista, Sweden) and an anesthetic conserving device (AnaConDa; Sedana Medical, Sweden). Continuous surveillance of anesthetic agent exchange and expiratory CO_2_ was monitored along with noninvasive blood pressure, temperature, heart rate, and pulse oximetry. 6-F introducers with hemostatic valves (Intradyn; B. Braun, Germany) were used for arterial and venous access through the femoral vessels with the Seldinger technique. Invasive arterial blood pressure was connected after successful arterial access. All invasive procedures were performed in a sterile manner, and 15,000 IE units of heparin was given after introducers were in place.

An infusion of 500 mL of crystalloid fluid with 300 mg of amiodarone was started ~30 min before ischemia-reperfusion to prevent arrhythmias. A 6-F coronary hydrophilic guiding catheter (Serpia; B. Braun, Germany) was used to perform a left coronary angiogram under fluoroscopy guidance. The appropriate location for balloon occlusion was obtained through a 0.014-in. guidewire. The occlusion was placed in the left anterior descending artery (LAD) at varied distances distal to the first diagonal (D1) depending on individual vessel anatomy but proximal to the second diagonal (D2). A percutaneous transluminal coronary angioplasty balloon (SeQuent NEO; B. Braun, Germany) was inflated with contrast solution, and obstruction of flow was confirmed with a subsequent angiogram. All pigs received an intravenous bolus of fentanyl (1 µg/kg) directly before occlusion. The duration of ischemia was 40 min before reperfusion. Reperfusion and return of coronary flow in distal LAD were confirmed through an additional coronary angiogram. The pigs were defibrillated (300 J) if they developed ventricular arrhythmias. Small intravenous boluses (5–15 µg) of norepinephrine were given if mean arterial pressure decreased below 50 mmHg. For pigs that were followed for additional scans, the sedation and anesthetic protocols were repeated. The animals were kept in housing in accordance with national regulations, had free access to water, and were examined daily by experienced personnel.

### Microembolization

Twelve healthy farm pigs (~33 kg) were premedicated with acepromazine (1.1 mg/kg) and ketamine (22–33 mg/kg) 30 min later and anesthetized with a mixture of isoflurane (2–5%) and oxygen (2–3 L/min). The pigs were intubated, and a 6-F introducer (Avanti; Cordis, Miami, FL) was placed in the femoral artery with the Seldinger technique. Selective coronary catheterization was performed under fluoroscopy guidance to locate and enter the LAD. Thereafter, a 3-F microcatheter (Cook, Chicago, IL) was positioned distal to the first diagonal (D1). The animal was then brought to the MRI scanner with the catheter in place.

The microembolic agent was delivered through the 3-F microcatheter in the pigs during a slow (60 s) infusion. A 60-min pause before the next imaging was then started to permit infarct development. The 12 pigs were divided into two subgroups of 6 pigs each, which in turn received two different sizes of microemboli (40–120 µm, 250,000 microsphere count and 100–300 µm, 7,200 microsphere count, respectively). The embolic characteristics for the two subgroups were calculated to approximately occlude the same volume of perfusion territory. Both 0.25-mL embolic solutions (Embosphere; Biosphere Medical, Rockland, MA) were diluted with 0.75 mL of NaCl solution.
